# 2-(2,5-Dioxotetra­hydro­furan-3-yl)isoindoline-1,3-dione

**DOI:** 10.1107/S1600536808024094

**Published:** 2008-08-06

**Authors:** Shao-Song Qian

**Affiliations:** a School of Life Sciences, Shandong University of Technology, Zibo 255049, People’s Republic of China

## Abstract

In the title compound, C_12_H_7_NO_5_, the dihedral angle between the isoindole-1,3-dione plane and the least-squares plane of the furan ring is 89.2 (2)°. In the crystal structure, mol­ecules are linked through inter­molecular C—H⋯O hydrogen bonds, forming centrosymmetric dimers.

## Related literature

For related literature, see: Abdel & Atef (2004 ▶); Allen *et al.* (1987 ▶); King & Kidd (1951 ▶); Qian *et al.* (2006 ▶).
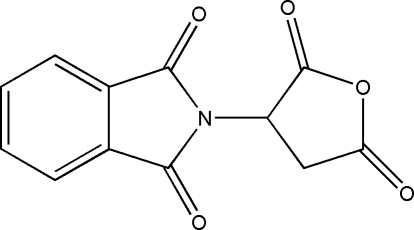


## Experimental

### 

#### Crystal data

C_12_H_7_NO_5_
*M* *_r_* = 245.19
Monoclinic, 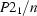

*a* = 12.129 (2) Å
*b* = 5.1385 (10) Å
*c* = 16.818 (3) Å
β = 100.21 (3)°
*V* = 1031.6 (4) Å^3^
*Z* = 4
Mo *K*α radiation
μ = 0.13 mm^−1^
*T* = 293 (2) K
0.30 × 0.30 × 0.05 mm

#### Data collection

Enraf–Nonius CAD-4 diffractometer
Absorption correction: ψ scan (*SADABS*; Sheldrick, 1996 ▶) *T*_min_ = 0.963, *T*_max_ = 0.994
1963 measured reflections
1870 independent reflections
1492 reflections with *I* > 2σ(*I*)
*R*_int_ = 0.040
3 standard reflections every 200 reflections intensity decay: none

#### Refinement

*R*[*F* ^2^ > 2σ(*F* ^2^)] = 0.043
*wR*(*F* ^2^) = 0.133
*S* = 1.07
1870 reflections
164 parameters
H-atom parameters constrained
Δρ_max_ = 0.31 e Å^−3^
Δρ_min_ = −0.21 e Å^−3^

### 

Data collection: *CAD-4 Software* (Enraf–Nonius, 1989 ▶); cell refinement: *CAD-4 Software*; data reduction: *XCAD4* (Harms & Wocadlo, 1995 ▶); program(s) used to solve structure: *SHELXS97* (Sheldrick, 2008 ▶); program(s) used to refine structure: *SHELXL97* (Sheldrick, 2008 ▶); molecular graphics: *XP* in *SHELXTL* (Sheldrick, 2008 ▶); software used to prepare material for publication: *SHELXL97*.

## Supplementary Material

Crystal structure: contains datablocks global, I. DOI: 10.1107/S1600536808024094/bt2757sup1.cif

Structure factors: contains datablocks I. DOI: 10.1107/S1600536808024094/bt2757Isup2.hkl

Additional supplementary materials:  crystallographic information; 3D view; checkCIF report

## Figures and Tables

**Table 1:** Hydrogen-bond geometry (Å, °)

*D*—H⋯*A*	*D*—H	H⋯*A*	*D*⋯*A*	*D*—H⋯*A*
C9—H9*A*⋯O1	0.98	2.54	2.915 (3)	103 (4)
C12—H12*B*⋯O5^i^	0.97	2.58	3.476 (3)	153 (4)

Symmetry code: (i) 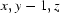.

**Fig. 1.**
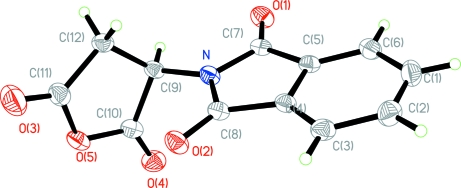
The structure of the title compound, showing the atom-labelling scheme. Displacement ellipsoids are drawn at the 35% probability level.

## supplementary crystallographic information

### Comment

The title compound  has attracted attention for its anticonvulsant activity (Abdel & Atef, 2004). In addition, it was an intermediate for the synthesis of aspartic acid (King & Kidd, 1951). Here, we report its crystal structure.

The dihedral angle between the isoindole-1,3-dione plane and the   plane of cyclopentane-1,3-dione is 90.0 (2)°. All the bond lengths are within normal ranges (Allen  *et al.*, 1987) and comparable to the values observed in other similar compounds (Qian  *et al.*, 2006). In the crystal structure, the molecules are linked through intermolecular C–H···O hydrogen bonds, forming centrosymmetric dimers.

### Experimental

The title compound   was synthesized according to a literature method (Qian *et al.*, 2006). *L*-aspartic acid (13.3 g, 0.1 mol) reacted with *N*-carboethoxy phthalimide (21.9 g, 0.1 mol) in 200 ml of water and 23.3 g (0.21 mol) of sodium carbonate. As a result, 21.3 g of the N-phthaloyl-*L*-aspartic acid was obtained (yield, 81%). 10.8 g of the title compound   was obtained through heating of N-phthaloyl-*L*-aspartic acid (13.2 g, 0.05 mol) in 30 ml of acetic anhydride under reflux for 20 minutes. Subsequently, 0.1 g of the title compound   was dissolved in acetic acid (20 ml). Single crystals suitable for X-ray diffraction were obtained by spontaneous evaporation of the solvent.

### Refinement

All   H atoms   were   geometrically positioned and constrained to ride on their parent atoms with C—H distance in the range 0.93–0.98 Å,   with *U*_iso_(H) = 1.2*U*_eq_(C).

### Crystal data

**Table:**

C_12_H_7_NO_5_	*F*(000) = 504
*M**_r_* = 245.19	*D*_x_ = 1.579 Mg m^−^^3^
Monoclinic, *P*2_1_/*n*	Mo *K*α radiation, λ = 0.71073 Å
Hall symbol: -P 2yn	Cell parameters from 25 reflections
*a* = 12.129 (2) Å	θ = 10–13°
*b* = 5.1385 (10) Å	µ = 0.13 mm^−^^1^
*c* = 16.818 (3) Å	*T* = 293 K
β = 100.21 (3)°	Prism, colorless
*V* = 1031.6 (4) Å^3^	0.30 × 0.30 × 0.05 mm
*Z* = 4	

### Data collection

**Table:**

Enraf–Nonius CAD-4 diffractometer	1492 reflections with *I* > 2σ(*I*)
Radiation source: fine-focus sealed tube	*R*_int_ = 0.040
graphite	θ_max_ = 25.3°, θ_min_ = 1.9°
ω/2θ scans	*h* = 0→14
Absorption correction: ψ scan (*SADABS*; Sheldrick, 1996)	*k* = 0→6
*T*_min_ = 0.963, *T*_max_ = 0.994	*l* = −20→19
1963 measured reflections	3 standard reflections every 200 reflections
1870 independent reflections	intensity decay: none

### Refinement

**Table:**

Refinement on *F*^2^	Secondary atom site location: difference Fourier map
Least-squares matrix: full	Hydrogen site location: inferred from neighbouring sites
*R*[*F*^2^ > 2σ(*F*^2^)] = 0.043	H-atom parameters constrained
*wR*(*F*^2^) = 0.133	*w* = 1/[σ^2^(*F*_o_^2^) + (0.0585*P*)^2^ + 0.7913*P*] where *P* = (*F*_o_^2^ + 2*F*_c_^2^)/3
*S* = 1.07	(Δ/σ)_max_ < 0.001
1870 reflections	Δρ_max_ = 0.31 e Å^−^^3^
164 parameters	Δρ_min_ = −0.21 e Å^−^^3^
0 restraints	Extinction correction: *SHELXL97* (Sheldrick, 2008), Fc^*^=kFc[1+0.001xFc^2^λ^3^/sin(2θ)]^-1/4^
Primary atom site location: structure-invariant direct methods	Extinction coefficient: 0.055 (5)

### Special details

**Table:**

Geometry. All e.s.d.'s (except the e.s.d. in the dihedral angle between two l.s. planes) are estimated using the full covariance matrix. The cell e.s.d.'s are taken into account individually in the estimation of e.s.d.'s in distances, angles and torsion angles; correlations between e.s.d.'s in cell parameters are only used when they are defined by crystal symmetry. An approximate (isotropic) treatment of cell e.s.d.'s is used for estimating e.s.d.'s involving l.s. planes.
Refinement. Refinement of *F*^2^ against ALL reflections. The weighted *R*-factor *wR* and goodness of fit *S* are based on *F*^2^, conventional *R*-factors *R* are based on *F*, with *F* set to zero for negative *F*^2^. The threshold expression of *F*^2^ > σ(*F*^2^) is used only for calculating *R*-factors(gt) *etc*. and is not relevant to the choice of reflections for refinement. *R*-factors based on *F*^2^ are statistically about twice as large as those based on *F*, and *R*- factors based on ALL data will be even larger.

### Fractional atomic coordinates and isotropic or equivalent isotropic displacement parameters (Å^2^)

**Table:**

	*x*	*y*	*z*	*U*_iso_*/*U*_eq_	
N	0.76293 (16)	−0.0485 (4)	0.04496 (11)	0.0347 (5)	
O1	0.88949 (15)	−0.3867 (4)	0.04821 (11)	0.0466 (5)	
C1	0.9239 (2)	0.0386 (6)	−0.18134 (16)	0.0487 (7)	
H1A	0.9710	0.0066	−0.2183	0.058*	
O2	0.65372 (15)	0.3125 (3)	0.00935 (10)	0.0430 (5)	
C2	0.8492 (2)	0.2458 (6)	−0.19425 (15)	0.0481 (7)	
H2A	0.8462	0.3474	−0.2404	0.058*	
C3	0.7791 (2)	0.3047 (5)	−0.14002 (14)	0.0409 (6)	
H3A	0.7292	0.4436	−0.1485	0.049*	
O3	0.44844 (18)	0.1302 (5)	0.13241 (15)	0.0729 (7)	
O4	0.81433 (16)	0.2349 (4)	0.20434 (11)	0.0523 (6)	
C4	0.78694 (19)	0.1471 (5)	−0.07286 (13)	0.0332 (6)	
O5	0.62761 (15)	0.2101 (3)	0.18451 (10)	0.0424 (5)	
C5	0.85913 (19)	−0.0636 (5)	−0.06052 (13)	0.0341 (6)	
C6	0.9295 (2)	−0.1217 (5)	−0.11412 (15)	0.0413 (6)	
H6A	0.9786	−0.2621	−0.1057	0.050*	
C7	0.84523 (19)	−0.1944 (5)	0.01570 (14)	0.0339 (6)	
C8	0.7244 (2)	0.1603 (5)	−0.00526 (13)	0.0338 (6)	
C9	0.7191 (2)	−0.1091 (5)	0.11716 (13)	0.0339 (6)	
H9A	0.7604	−0.2566	0.1449	0.041*	
C10	0.7312 (2)	0.1266 (5)	0.17350 (14)	0.0388 (6)	
C11	0.5426 (2)	0.0628 (6)	0.13833 (15)	0.0445 (7)	
C12	0.5937 (2)	−0.1667 (5)	0.10354 (15)	0.0388 (6)	
H12A	0.5639	−0.1847	0.0464	0.047*	
H12B	0.5791	−0.3258	0.1309	0.047*	

### Atomic displacement parameters (Å^2^)

**Table:**

	*U*^11^	*U*^22^	*U*^33^	*U*^12^	*U*^13^	*U*^23^
N	0.0446 (11)	0.0297 (10)	0.0303 (10)	0.0057 (9)	0.0077 (8)	0.0006 (8)
O1	0.0528 (11)	0.0376 (10)	0.0481 (11)	0.0136 (9)	0.0056 (8)	0.0040 (8)
C1	0.0487 (15)	0.0611 (18)	0.0394 (14)	−0.0068 (14)	0.0165 (12)	−0.0077 (14)
O2	0.0536 (11)	0.0369 (10)	0.0398 (10)	0.0133 (9)	0.0117 (8)	0.0027 (8)
C2	0.0594 (17)	0.0513 (17)	0.0336 (13)	−0.0100 (14)	0.0082 (12)	0.0012 (12)
C3	0.0535 (15)	0.0341 (13)	0.0339 (13)	−0.0014 (12)	0.0045 (11)	−0.0007 (11)
O3	0.0529 (13)	0.0827 (17)	0.0840 (16)	0.0099 (13)	0.0147 (11)	−0.0248 (14)
O4	0.0595 (12)	0.0508 (12)	0.0444 (11)	−0.0101 (10)	0.0036 (9)	−0.0093 (9)
C4	0.0392 (12)	0.0296 (12)	0.0302 (12)	−0.0021 (11)	0.0046 (10)	−0.0041 (10)
O5	0.0583 (11)	0.0337 (10)	0.0353 (9)	0.0070 (8)	0.0090 (8)	−0.0050 (7)
C5	0.0388 (12)	0.0292 (12)	0.0328 (12)	−0.0040 (10)	0.0023 (10)	−0.0057 (10)
C6	0.0406 (13)	0.0396 (14)	0.0432 (14)	0.0004 (12)	0.0061 (11)	−0.0071 (12)
C7	0.0367 (12)	0.0276 (12)	0.0351 (12)	0.0009 (11)	0.0001 (10)	−0.0051 (10)
C8	0.0411 (13)	0.0285 (12)	0.0309 (12)	0.0018 (11)	0.0036 (10)	−0.0012 (10)
C9	0.0468 (14)	0.0266 (12)	0.0287 (11)	0.0047 (11)	0.0073 (10)	0.0014 (10)
C10	0.0555 (16)	0.0324 (13)	0.0277 (12)	0.0030 (12)	0.0048 (11)	0.0021 (10)
C11	0.0523 (16)	0.0446 (16)	0.0386 (14)	0.0034 (13)	0.0135 (12)	−0.0014 (12)
C12	0.0505 (15)	0.0286 (13)	0.0386 (13)	−0.0008 (11)	0.0110 (11)	0.0002 (10)

### Geometric parameters (Å, °)

**Table:**

N—C8	1.394 (3)	C4—C5	1.385 (3)
N—C7	1.405 (3)	C4—C8	1.476 (3)
N—C9	1.443 (3)	O5—C10	1.370 (3)
O1—C7	1.208 (3)	O5—C11	1.398 (3)
C1—C6	1.391 (4)	C5—C6	1.380 (3)
C1—C2	1.390 (4)	C5—C7	1.483 (3)
C1—H1A	0.9300	C6—H6A	0.9300
O2—C8	1.217 (3)	C9—C12	1.526 (3)
C2—C3	1.387 (4)	C9—C10	1.529 (3)
C2—H2A	0.9300	C9—H9A	0.9800
C3—C4	1.379 (3)	C11—C12	1.499 (4)
C3—H3A	0.9300	C12—H12A	0.9700
O3—C11	1.180 (3)	C12—H12B	0.9700
O4—C10	1.188 (3)		
			
C8—N—C7	112.40 (19)	O1—C7—C5	130.7 (2)
C8—N—C9	122.82 (19)	N—C7—C5	104.9 (2)
C7—N—C9	124.76 (19)	O2—C8—N	123.0 (2)
C6—C1—C2	121.1 (2)	O2—C8—C4	131.4 (2)
C6—C1—H1A	119.4	N—C8—C4	105.6 (2)
C2—C1—H1A	119.4	N—C9—C12	114.92 (19)
C3—C2—C1	121.6 (2)	N—C9—C10	109.97 (19)
C3—C2—H2A	119.2	C12—C9—C10	103.30 (19)
C1—C2—H2A	119.2	N—C9—H9A	109.5
C4—C3—C2	116.7 (2)	C12—C9—H9A	109.5
C4—C3—H3A	121.7	C10—C9—H9A	109.5
C2—C3—H3A	121.7	O4—C10—O5	121.5 (2)
C3—C4—C5	122.1 (2)	O4—C10—C9	128.5 (2)
C3—C4—C8	129.3 (2)	O5—C10—C9	110.0 (2)
C5—C4—C8	108.6 (2)	O3—C11—O5	119.7 (3)
C10—O5—C11	111.05 (19)	O3—C11—C12	131.2 (3)
C6—C5—C4	121.3 (2)	O5—C11—C12	109.1 (2)
C6—C5—C7	130.2 (2)	C11—C12—C9	105.0 (2)
C4—C5—C7	108.5 (2)	C11—C12—H12A	110.7
C5—C6—C1	117.2 (2)	C9—C12—H12A	110.7
C5—C6—H6A	121.4	C11—C12—H12B	110.7
C1—C6—H6A	121.4	C9—C12—H12B	110.7
O1—C7—N	124.3 (2)	H12A—C12—H12B	108.8
			
C6—C1—C2—C3	1.4 (4)	C9—N—C8—C4	−177.8 (2)
C1—C2—C3—C4	−0.2 (4)	C3—C4—C8—O2	−1.9 (4)
C2—C3—C4—C5	−1.5 (4)	C5—C4—C8—O2	179.3 (3)
C2—C3—C4—C8	179.9 (2)	C3—C4—C8—N	178.5 (2)
C3—C4—C5—C6	1.9 (4)	C5—C4—C8—N	−0.3 (3)
C8—C4—C5—C6	−179.2 (2)	C8—N—C9—C12	59.4 (3)
C3—C4—C5—C7	−178.7 (2)	C7—N—C9—C12	−118.4 (2)
C8—C4—C5—C7	0.2 (3)	C8—N—C9—C10	−56.6 (3)
C4—C5—C6—C1	−0.7 (4)	C7—N—C9—C10	125.6 (2)
C7—C5—C6—C1	−179.9 (2)	C11—O5—C10—O4	176.2 (2)
C2—C1—C6—C5	−1.0 (4)	C11—O5—C10—C9	−2.7 (3)
C8—N—C7—O1	−178.7 (2)	N—C9—C10—O4	−61.0 (3)
C9—N—C7—O1	−0.7 (4)	C12—C9—C10—O4	175.9 (3)
C8—N—C7—C5	−0.2 (3)	N—C9—C10—O5	117.8 (2)
C9—N—C7—C5	177.9 (2)	C12—C9—C10—O5	−5.3 (2)
C6—C5—C7—O1	−2.3 (4)	C10—O5—C11—O3	−170.0 (3)
C4—C5—C7—O1	178.4 (2)	C10—O5—C11—C12	9.9 (3)
C6—C5—C7—N	179.3 (2)	O3—C11—C12—C9	167.1 (3)
C4—C5—C7—N	0.0 (2)	O5—C11—C12—C9	−12.7 (3)
C7—N—C8—O2	−179.4 (2)	N—C9—C12—C11	−109.3 (2)
C9—N—C8—O2	2.6 (4)	C10—C9—C12—C11	10.5 (2)
C7—N—C8—C4	0.3 (3)		

### Hydrogen-bond geometry (Å, °)

**Table:**

*D*—H···*A*	*D*—H	H···*A*	*D*···*A*	*D*—H···*A*
C9—H9A···O1	0.98	2.54	2.915 (3)	103 (4)
C12—H12B···O5^i^	0.97	2.58	3.476 (3)	153 (4)

Symmetry codes: (i) *x*, *y*−1, *z*.
